# Clinical characterization of neuropathic pain and small fiber impairment in neurofibromatosis

**DOI:** 10.1097/PR9.0000000000001285

**Published:** 2025-05-06

**Authors:** Eva Meller, Annsophie Amann, Rayaz A. Malik, Daniel Kampik, Cordula Matthies, Nurcan Üçeyler

**Affiliations:** Departments of a Neurosurgery and; b Neurology, University Hospital Würzburg, Würzburg, Germany; c Weill Cornell Medicine-Qatar, Ar Rayyān, Doha, Qatar; d Departement of Ophthalmology, University Hospital Würzburg, Würzburg, Germany

**Keywords:** Neurofibromatosis, Neuropathic pain, Small fiber neuropathy, Skin punch biopsy

## Abstract

**Introduction:**

Pain is an often underestimated symptom in patients with neurofibromatosis (NF) and schwannomatosis (SWN), yet it may have a profound impact on health-related quality of life.

**Objective:**

To assess the characteristics of neuropathic pain and small nerve fiber impairment in patients with NF.

**Methods:**

In this case-control study, we enrolled 51 patients who fulfilled the diagnostic criteria for neurofibromatosis type 1 (NF1), NF2-related schwannomatosis (SWN-NF2), and schwannomatosis-not otherwise specified (SWN-NOS). Patients completed validated questionnaires for pain and health-related quality of life and underwent neurological examination, nerve conduction studies, nerve sonography, quantitative sensory testing (QST), distal and proximal skin-punch biopsy, and corneal confocal microscopy (CCM).

**Results:**

Pain was reported by 28 of 51 (55%) patients with 18 of 51 (35%) suffering from neuropathic pain. Pain was chronic (≥3 months) in all patients with neuropathic pain. In 28 of 51 (55%) patients, skin biopsies revealed signs of denervation, while 7 of 51 (14%) patients exhibited ≥2 pathological small fiber tests and were classified as having small fiber impairment.

**Conclusion:**

Neuropathic pain and small fiber abnormalities are prevalent among patients with NF as well as SWN. Although no clear correlation between small fiber damage and painful neuropathy was found, these findings need special attention and correct categorization to offer efficient analgesic treatment.

## Abstract 2

Neuropathic pain and small fiber impairment are common in neurofibromatosis, impairing quality of life. Accurate categorization and treatment are crucial for effective pain management.

## 1. Introduction

Neurofibromatosis (NF) comprises a group of genetic disorders with benign peripheral nerve tumors due to excessive proliferation of Schwann cells. The 3 subtypes NF1, SWN-NF2, and SWN-NOS are due to distinct variants in tumor suppressor genes encoding neurofibromin (NF1), merlin (SWN-NF2), and variants in other related genes (such as leucine-zipper-like transcriptional regulator 1 [LZTR1] gene, SWI/SNF-related matrix-actin-dependent regulator of chromatin subfamily B member 1 [SMARCB1] gene).^12,14^ The clinical manifestations and tumor growth patterns of NF are diverse.^15,20^ Pain in NF can range from localized discomfort at the site of neurofibromas or schwannomas to severe neuropathic pain from nerve entrapment or compression.^3^ NF pain may, however, also occur in the absence of compressing tumors and the underlying pathophysiology remains unclear.^34^ Polyneuropathy in patients with NF1 is rare,^6,11^ but peripheral neuropathy with sensory loss and impaired nerve conduction studies is frequent in patients with SWN-NF2.^26^ Painful neuropathy is a feature of SWN-NOS, and recent studies suggest the involvement of C-fibers.^10^ Hence, neuronal hypersensitivity may occur independent of tumor formation.^6^

Previous studies on NF-related neuropathy focused primarily on identifying typical symptoms and signs and nerve conduction abnormalities attributed to compression from nerve tumors.^11,26^ However, there may be a number of potential mechanisms to explain the emergence of neuropathic pain. Injury to Schwann cells due to tumors could trigger increased production of proinflammatory and algesic cytokines.^18^ Schwann cells affected by tumors may reduce the secretion of neurotrophic factors, hindering physiological axonal sprouting.^1^ Very few studies have assessed the involvement of small nerve fibers in NF,^2,24^ even though the neuropathy of NF has many of the features of small fiber neuropathy (SFN) such as burning pain, par-/dysesthesias, hyperalgesia, allodynia, and hyposensitivity to thermal stimuli.^23^

In this cross-sectional study, we aimed to investigate the prevalence of neuropathic pain and small fiber impairment (SFI) in patients with NF and hypothesized that subgroups of patients with NF experience small fiber dysfunction that is not linked to focal nerve compression.

## 2. Methods

### 2.1. Study participants

Between February 2021 and November 2023, we recruited adult patients with NF1, SWN-NF2- and SWN-NOS who met the respective clinical diagnostic criteria and were seen at the Department of Neurosurgery, University Hospital Würzburg, Germany.^15,20^ Exclusion criteria for patients included ongoing infectious disease, substance abuse, insufficient physical fitness to participate in the examinations, and inability to understand and fill in the questionnaires. Patient data were compared with data of age- and sex-matched adult healthy controls individually collected for the respective analysis. Inclusion criteria for healthy controls were no neuropathic pain, with neuropathy ruled out by clinical examination and sensory neurography of the sural nerve. Our study was approved by the Ethics Committee of the University of Würzburg Medical Faculty, Germany (#136/20) and was carried out in accordance with the Declaration of Helsinki. All participants provided written informed consent before inclusion.

### 2.2. Clinical examination and questionnaire assessment

A medical history, detailed individual pain interview, and complete neurological examination were performed for all patients. We defined 4 categories: neuropathic pain not caused by tumor (NP_øTU_), neuropathic pain caused by tumor (NP_TU_), pain other than neuropathic (P_øNP_), and no pain (øP). Neuropathic pain was defined according to the International Association for the Study of Pain (IASP) as “pain that arises as a direct consequence of a lesion or disease affecting the somatosensory system,” following the Neuropathic Pain Special Interest Group (NeuPSIG) grading system.^23^ The diagnosis of SFI was based on the diagnostic criteria for SFN, requiring the presence of clinical symptoms along with at least 2 pathological findings in small fiber tests (eg, reduced intraepidermal nerve fiber density) and/or abnormal results in functional small fiber assessments. However, unlike SFN, SFI was defined in patients who did not exhibit the characteristic acral burning pain. To assess the link between pain and the presence of a nerve tumor, previously diagnostic MRI scans of the brain, spinal cord, and peripheral nervous system were reviewed. All patients had recent MRI imaging, and the relevant body regions were imaged after the onset of neuropathic pain. For the assessment of pain intensity, we used the numeric rating scale (NRS) and patients were asked to rate individual pain on a scale from 0 to 10, with 0 indicating no pain and 10 indicating the worst pain imaginable. Patients further filled in 2 validated pain questionnaires: (1) the German version of the Graded Chronic Pain Scale (GCPS)^32^ quantifying pain severity and disability associated with chronic pain; (2) the Neuropathic Pain Symptom Inventory (NPSI) which comprehensively characterizes neuropathic pain.^4,25^ In addition, the 12-item Short Form Survey (SF-12) was used to assess health-related quality of life.

### 2.3. Large nerve fiber assessment

#### 2.3.1. Nerve conduction studies

Sensory nerve conduction studies (NCSs) of the right median and sural nerves and motor NCS and F-wave studies of the right median and tibial nerve were undertaken following standard procedures. For the assessment of large fiber function, the sural sensory nerve action potential (SNAP) amplitude and nerve conduction velocity (NCV) were considered as the main outcome variables. Results were compared with the reference values from the neurological department's electroneurography unit. The limit for sural nerve SNAP amplitude was 10 μV for patients ≤65 years and 5 μV for those >65 years; normal sural nerve NCV was above 40 m/second for all adults. For the tibial nerve, the CMAP should be ≥10 mV, with a distal motor latency upper limit of 6.0 ms and a lower limit of NCV of 40 m/second.

#### 2.3.2. High-resolution ultrasonography

Median nerve sonography was performed as an additional test for large nerve fiber involvement, providing dynamic assessment to complement neurological examination, nerve conduction studies, and MRI and to detect early subclinical alterations with potential clinical significance. Patients underwent high-resolution ultrasonography of the right median nerve at a standardized location. The Aplio XG ultrasonography device (Toshiba Medical Systems, STADT, Japan) with an 18-MHz linear transducer and its standard Tissue Harmonic Imaging Software was utilized. The evaluation was performed following a standardized protocol for a transverse plane with patients positioned prone, and measurements of cross-sectional area (CSA) were performed at the wrist, mid-forearm, cubital fossa, and mid-upper arm. Throughout the assessments, the device mode remained constant and measurements were taken without utilizing the zoom function.

### 2.4. Small nerve fiber tests

#### 2.4.1. Quantitative sensory testing

Following the standardized protocol of the German Research Network on Neuropathic Pain (Deutscher Forschungsverbund Neuropathischer Schmerz, DFNS e.V.),^22^ Quantitative sensory testing (QST) was performed on the dorsum of the right foot using a calibrated device (Somedic, Hörby, Sweden). The results were compared individually with published reference data.^16^ To better align with the demographic characteristics of our patient cohort, we further compared data with an age- and sex-matched control cohort comprising 60 healthy individuals (median age 42 years, range 21–70), all examined in the QST laboratory of the Department of Neurology, University Hospital Würzburg. A z-score was calculated by log transformation of the raw values. A negative z-score was interpreted as loss-of-function and a positive z-score as gain-of-function.

#### 2.4.2. Skin innervation

To assess the intraepidermal nerve fiber density (IENFD), a skin-punch biopsy was performed on the right lateral lower leg and upper thigh using a 6-mm circular skin-punch instrument following a standardized procedure.^31^ For the determination of IENFD, nerve fibers within the epidermis were visualized by immunoreaction with antibodies against the protein-gene product (PGP) 9.5 (Ultraclone, United Kingdom, 1:800; primary antibody) with a secondary goat anti-rabbit IgG antibody labelled with cyanine 3 (Cy3) (Amersham, 1:100; Cy3, secondary antibody). Nerve fibers were counted by an observer blinded to subject allocation in accordance with a published protocol.^13^ Immunohistochemical staining for CD3 (T cells) and CD68 (macrophages) was routinely performed on diagnostic skin-punch biopsies, to evaluate potential local inflammatory processes contributing to neuropathic pain. The presence of dermal T cells and macrophages was qualitatively determined by immunohistochemical staining using antibodies against the surface markers CD3 (Abcam, Germany, 1:200; primary antibody) and CD68 (Abcam, 1:3000, primary antibody). Using our laboratory normative values (distal IENFD: 9 fibers/mm ± 3 fibers/mm; proximal IENFD: 12 fibers/mm ± 4 fibers/mm), we defined pathological nerve fiber density as <6 fibers/mm at the lower leg and <8 fibers/mm at the upper thigh. The control group consisted of 31 women and 28 men (women: median age 38 years, range 20–56; men: median age 44 years, range 23–75).

#### 2.4.3. Corneal confocal microscopy

After ruling out corneal pathology through slit-lamp examination by an ophthalmologist, corneal confocal microscopy (CCM) was performed on both eyes of each study participant using the Heidelberg Retina Tomograph Rostock Cornea Module (Heidelberg Engineering, Heidelberg, Germany).^29^ Images of the central subbasal nerve plexus were analyzed for nerve fiber density (NFD), nerve fiber length (NFL), and nerve branch density (NBD). To assess whether pathological values were present, we also compared our data with published normative values for CCM.^28^ The control group consisted of 29 women and 21 men (women: median age 38 years, range 22–57, men: median age 43 years, range 21–68). Corneal confocal microscopy results were primarily used to assess SFI more generally, rather than focusing on specific nerve damage. This broader approach provided insights into generalized SFI across the entire patient cohort. The inclusion of CCM data in the overall evaluation allowed for a more comprehensive understanding of the role of generalized small fiber dysfunction in the context of neuropathic pain.

### 2.5. Statistical analysis

We conducted statistical analyses using SPSS 26 software (IBM, Ehningen, Germany). The main outcome variables of the study were SFI, assessed by IENFD, CCM parameters, QST, and pain scores. Data distribution was evaluated using the Shapiro-Wilk test. For nonnormally distributed data, we employed the nonparametric Mann-Whitney *U* test, with results presented as median values and ranges. Categorical data comparisons were performed using the Chi^2^ test to evaluate differences between predefined groups. These groups were categorized by pain type (no pain, nonneuropathic pain, neuropathic pain with tumor association, and neuropathic pain without tumor association) as well as by NF subtype (eg, NF1, NF2, SWN). A *P*-value <0.05 was considered statistically significant. To assess the relationships between demographic and clinical characteristics (eg, age, sex, NF/SWN subtype) and the outcome variables, one-way analyses of variance (ANOVAs) were conducted. These analyses specifically evaluated whether differences in pain categories or NF subtypes were associated with variations in pain intensity, pain-related impairment, and the physical and mental component scores of the SF-12.

## 3. Results

### 3.1. Pain history and clinical characterization

**Figure 1.**
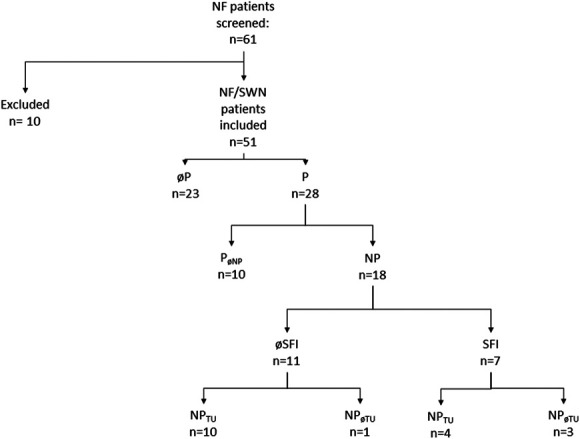
Patient stratification. The algorithm shows how study participants were stratified with regard to pain and small nerve fiber pathology. NF, neurofibromatosis; NP, neuropathic pain; NPøTU, neuropathic pain not caused by tumor; NPTU, neuropathic pain caused by tumor; P, pain; PøNP, pain other than neuropathic; øP, no pain; n, number; SFI, small fiber impairment.

Details of the study cohort in relation to pain descriptors and accompanying symptoms are provided in Table 1. We screened 61 patients and included 51 patients in this prospective case-control study (24 men; median age 37 years, range 18–75). Ten patients were excluded due to lack of physical fitness (3/61; 5%) or withdrawal of consent before the start of study procedures (7/61; 11%). Our study cohort of 51 patients consisted of 33 patients with NF1, 11 with SWN-NF2, and 7 with SWN-NOS (Fig. 1). Overall, 28 of 51 (55%) patients reported pain, with a median pain intensity of ≥3 on the numeric rating scale (NRS) during the 10 weeks before the examination (range 3–10). All 28 patients with pain (100%) had chronic pain, defined as persisting for ≥3 months. Among these patients, 20 of 28 (71%) required regular pain medication.

**Table 1:** Demographic data and pain characteristics of patient cohort.

NF patients (N)	51
M, F (N)	24, 27
NF1 (N)	33
SWN-NF2 (N)	11
SWN-NOS (N)	7
Median age (range) (y)	37 (18–75)
Patients with pain (N, %) NF1 SWN-NF2 SWN-NOS	28/51 (55%) 16/33 (48%) 6/11 (54%) 6/7 (86%)
Pain subgroups (N, %) NP_øTU_ NP_TU_ P_øNP_ øP	4/51 (8%) 14/51 (27%) 10/51 (20%) 23/51 (45%)
Pain medication (N, %) NF1 SWN-NF2 SWN-NOS	20/51 (39%) 12/33 (36%) 4/11 (36%) 4/7 (57%)
Duration of pain ≥1 y (N, %)	28/28 (100%)

F, female; M, male; N, number; NF, neurofibromatosis; NP_øTU_, neuropathic pain not caused by tumor; P_TU_, neuropathic pain caused by tumor; P_øNP_, pain other than neuropathic; øP, no pain; SWN-NF2, NF2-related schwannomatosis; SWN-NOS, schwannomatosis—not otherwise specified.

**Figure 2.**
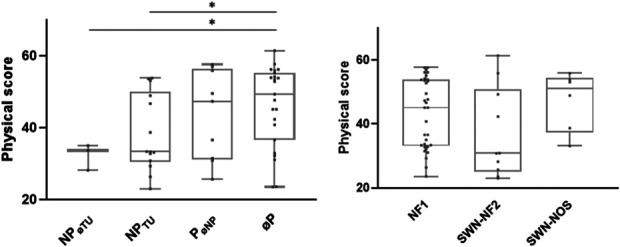
Association between pain and physical activity. (A) Physical score (SF-12) pain type. (B) Physical score (SF-12) and NF type. Patients with neuropathic pain unrelated to tumors reached a lower physical score in the SF-12. Scores did not differ between tumor types. (1) Neuropathic pain no tumor; (2) neuropathic pain tumor; (3) other pain; (4) no pain GCPS graded chronic pain scale. NF, neurofibromatosis; SF-12, Short-Form-Health Survey 12.

**Figure 3.**
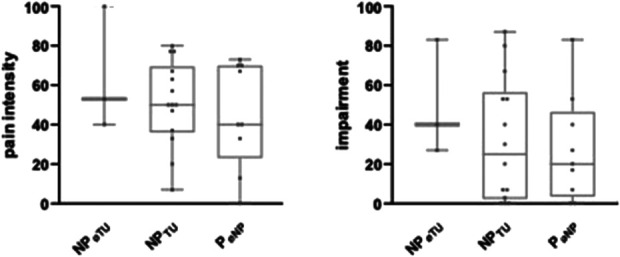
Pain intensities stratified for pain types. No differences were observed in (A) pain intensity and (B) subjective impairment due to pain when stratified for pain type and neuropathic pain type. (1) Neuropathic pain no tumor; (2) neuropathic pain tumor; (3) other pain; (4) no pain GCPS graded chronic pain scale. NF, neurofibromatosis.

Age, sex, NF type, or pain type showed no influence on pain intensity or impairment due to pain. In the SF-12, the physical health score (mean 37 ± 10) was lower in the pain group compared with patients with no pain (*P* < 0.05). However, there was no difference between the 2 groups in terms of mental health scores (mean 40 ± 13). The results of the NPSI, SF-12, and GCPS are presented in Table 2 as well as Figures 2 and 3.

**Table 2:** Analysis of questionnaire data on pain and health-related quality of life.

NPSI	NF/SWN type		Pain subgroup	
Sum score	NF1 SWN-NF2 SWN-NOS	11 (0–36) 9 (0–15) 11 (1–19)	NP_øTU_ NP_TU_ P_øNP_ øP	18 (16–24) 16 (1–28) 12 (2–36) 6 (0–30)
Burning score	NF1 SWN-NF2 SWN-NOS	2 (0–9) 2 (0–10) 2 (0–8)	NP_øTU_ NP_TU_ P_øNP_ øP	6 (3–10) 3 (0–8) 3 (0–9) 1 (0–5)
Pressure score	NF1 SWN-NF2 SWN-NOS	3 (0–9) 3 (0–7) 2 (0–5)	NP_øTU_ NP_TU_ P_øNP_ øP	4 (2–5) 3 (0–9) 3 (0–8) 2 (0–8)
Number of attacks	NF1 SWN-NF2 SWN-NOS	3 (0–9) 2 (0–9) 3 (0–8)	NP_øTU_ NP_TU_ P_øNP_ øP	6 (3–9) 4 (0–6) 3 (0–9) 1 (0–8)
Evoked pain score	NF1 SWN-NF2 SWN-NOS	2 (0–8) 1 (0–5) 2 (0–3)	NP_øTU_ NP_TU_ P_øNP_ øP	1 (0–1) 3 (0–6) 2 (0–8) 1 (0–5)
Par-/dysesthesia score	NF1 SWN-NF2 SWN-NOS	2 (0–8) 1 (0–6) 2 (0–6)	NP_øTU_ NP_TU_ P_øNP_ øP	2 (0–5) 3 (0–8) 2 (0–8) 1 (0–8)

SF12	NF/SWN type		Pain subgroup	
Physical score	NF1 SWN-NF2 SWN-NOS	43 (26–58) 37 (23–62) 47 (33–56)	NP_øTU_ NP_TU_ P_øNP_ øP	32 (28–35) 38 (23–54) 44 (26–58) 45 (24–61)
Mental score	NF1 SWN-NF2 SWN-NOS	46 (22–60) 38 (24–56) 44 (15–61)	NP_øTU_ NP_TU_ P_øNP_ øP	33 (24–41) 40 (22–61) 43 (22–56) 48 (23–61)

GCPS	NF/SWN type		Pain subgroup	
Subjective impairment	NF1 SWN-NF2 SWN-NOS	19 (0–87) 30 (0–83) 20 (0–67)	NP_øTU_ NP_TU_ P_øNP_ øP	50 (27–100) 32 (0–87) 27 (0–83) 8 (0–33)
Pain intensity	NF1 SWN-NF2 SWN-NOS	31 (0–80) 43 (0–100) 41 (0–77)	NP_øTU_ NP_TU_ P_øNP_ øP	64 (40–100) 51 (7–80) 45 (0–73) 15 (0–43)

Median scores and standard deviations are given for NPSI, GCPS, and SF12 data.

GCPS, graded chronic pain scale; NF, neurofibromatosis; NP_øTU_, neuropathic pain not caused by tumor; NPSI, neuropathic pain symptom inventory; P_TU_, neuropathic pain caused by tumor; P_øNP_, pain other than neuropathic; øP, no pain; SF-12, Short-Form-Health Survey 12; SWN-NF2, NF2-related schwannomatosis; SWN-NOS, schwannomatosis—not otherwise specified.

### 3.2. Pain classification

Neurological examination and assessment of previously performed MRI studies revealed that 18 of 28 (64%) patients with pain had a lesion or disease of the somatosensory nervous system, indicating neuropathic pain. Nerve conduction studies were performed in 41 of 51 (80%) patients, revealing abnormalities in 15 of 41 (37%) patients. Pain was directly attributable to a compressing nerve tumor in 14 of 18 (78%) of these patients. Therefore, 14 of 51 (27%) of the overall cohort experienced neuropathic pain related to a tumor. In addition to tumor-related neuropathic pain, 4 of 51 (8%) patients had neuropathic pain not associated with a tumor, identified by the clinical and diagnostic assessments, such as nerve conduction studies and lesion locations. In total, 10 of 51 (20%) participants experienced pain that could not be classified as neuropathic, including nonspecific back pain (3 cases) and migraine (1 case). In none of our pain subgroups, we observed the typical acral burning pain associated with small fiber neuropathy. Table 3 summarizes the clinical characteristics of patients with neuropathic pain.

**Table 3:** Patients with NF or SWN and neuropathic pain.

Patient ID	Age (yrs)	Sex	NF/SWN type	Pain location	SFP	Pain-inducing tumor
NF01	55	M	SWN-NF2	Whole body	Yes	—
NF02	37	F	SWN-NF2	Head, back, right thigh	No	Multiple intracranial and spinal schwannoma and meningioma
NF05	20	F	SWN-NOS	Left arm and hand	No	Multiple schwannoma left arm
NF07	59	M	1	Left arm and hand	No	Multiple neurofibroma left arm
NF13	72	M	1	Right leg	No	Neurofibroma right sciatic nerve
NF18	52	F	SWN-NOS	Right leg	No	Schwannoma right tibial nerve
NF21	31	F	SWN-NF2	Face	No	Multiple intracranial schwannoma and meningioma
NF23	24	M	1	Left leg	No	Neurofibroma left sural nerve
NF24	57	M	1	Neck, abdomen	Yes	Neurofibroma cervical spine
NF27	24	F	1	Right thigh	No	Cutaneous plexiform neurofibroma right thigh
NF28	42	M	1	Shoulders, neck, legs	Yes	—
NF40	42	M	SWN-NOS	Hands, feet	No	—
NF41	44	F	1	Right arm, legs, back	Yes	Neurofibroma cervical spine, multiple cutaneous neurofibroma
NF43	60	F	1	Back, legs	Yes	—
NF46	18	M	1	Left foot	No	Plexiform neurofibroma left foot
NF48	43	M	SWN-NOS	Right leg	Yes	Multiple schwannoma right leg
NF50	46	F	SWN-NOS	Right leg	Yes	Multiple schwannoma right leg
NF59	42	M	SWN-NOS	Left foot	No	Multiple schwannoma left leg

F, female; M, male; NF, neurofibromatosis; SFP, small fiber pathology; SWN, schwannomatosis; SWN-NF2, NF2-related schwannomatosis; SWN-NOS, schwannomatosis—not otherwise specified.

In total, 7 of 18 (39%) patients with neuropathic pain also met our criteria for SFI.^5^ Distribution among the patient groups was 4 NF1, 1 SWN-NF2, and 2 SWN-NOS. The prevalence of SFI was particularly high in the NPTU group. Four patients in the NPTU group and 3 patients in the NPøTU group met the criteria for SFI.

Among the 18 patients with neuropathic pain, 6 of 18 (33%) reported a high degree of disability (GCPS grade >3), with significant impairment in daily functioning due to pain. This was similar to the 2 of 10 (20%) patients with nonneuropathic pain who also reached a grade >3 on the GCPS. There were no significant differences between the NPøTU group and the NPTU group regarding pain-related disability.

### 3.3. Abnormalities in small fiber function and morphology in patients with neurofibromatosis

Quantitative sensory testing was performed on the back of the right foot in all 51 patients. A group-wise comparison of QST data from our patients and healthy control subjects revealed that the mechanical pain threshold (MPT) was elevated in all patients with NF compared with the healthy control group (NF1 *P* < 0.001; SWN-NF2 *P* < 0.001; SWN-NOS *P* < 0.01). Patients with SWN-NF2 also showed an elevated mechanical detection threshold (MDT) (*P* < 0.01), while patients with SWN-NOS had elevated mechanical pain sensitivity (MPS) (*P* < 0.05). The cold detection threshold (CDT) was higher in patients with neuropathic pain (*P* < 0.05).

**Figure 4.**
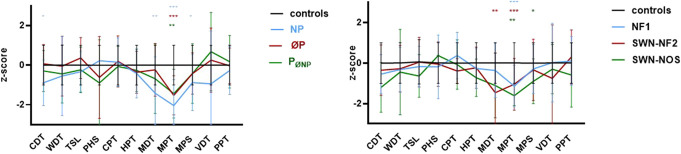
Sensory profiles. (A) QST profiles stratified for pain with elevated CDT in patients suffering from neuropathic pain (*P* < 0.05). (B) QST profiles stratified for disease subgroup with elevated MPT in all NF types compared with the healthy control group (NF1 *P* < 0.001; SWN-NF2 *P* < 0.001; SWN-NOS *P* < 0.01). Elevated MDT (*P* < 0.01) in patients with NF2 and elevated MPS in patients with SWN-NOS (*P* < 0.05) compared with the healthy control group. CDT, cold detection threshold; CPT, cold pain threshold; HPT, heat pain threshold; MDT, mechanical detection threshold; MPT, mechanical pain threshold; MPS, mechanical pain sensitivity; NF, neurofibromatosis; NP, neuropathic pain; PøNP, pain other than neuropathic; øP, no pain; PHS, paradoxical heat sensation; PPT, pressure pain threshold; QST, quantitative sensory testing; SWN-NOS, schwannomatosis—not otherwise specified, NF2-related schwannomatosis (SWN-NF2); TSL, thermal sensory limen VDT vibration detection threshold, WDT warm detection threshold.

When comparing QST profiles of patients with NF and SWN with reference values,^16^ we found that 6 of 51 (12%) patients exhibited an elevated CDT, 7 of 51 (14%) had an elevated warm detection threshold (WDT), and 9 of 51 (18%) showed an elevated thermal sensory limen (TSL). The MDT was elevated in 10 of 51 (20%) patients (Fig. 4). Since the test area was always the right foot, QST alterations were typically observed as generalized changes, rather than being localized to the neuro-anatomical distribution of a specific nerve, regardless of tumor involvement.

Corneal confocal microscopy was performed in 45 of 51 (88%) patients. When compared with published normative values,^28^ corneal innervation was reduced in 6 of 31 (19%) patients with NF1, 5 of 9 (55%) with SWN-NF2, and 0 of 5 with SWN-NOS (Table 4).

**Table 4:** Corneal and intraepidermal innervation.

	CCM			IENFD	
	NFD	NBD	NFL	Distal	Proximal
NF1	0/31	1/31 (3%)	6/31 (19%)	15/32 (47%)	6/31 (19%)
SWN-NF2	2/9 (22%)	3/9 (33%)	4/9 (44%)	7/11 (64%)	4/11 (36%)
SWN-NOS	0/5	0/5	0/5	4/7 (57%)	1/6 (17%)
NP_øTU_	1/4 (25%)	1/4 (25%)	1/4 (25%)	2/4 (50%)	3/4 (75%)
NP_øTU_	0/11	3/11 (27%)	3/11 (27%)	4/13 (31%)	9/14 (64%)
P_øNP_	1/8 (13%)	1/8 (13%)	4/8 (50%)	2/10 (20%)	3/10 (30%)
øP	0/22	1/22 (5%)	3/22 (14%)	2/21 (14%)	11/22 (50%)

Percentage of patients with CCM and IENFD data falling below the age- and sex-matched bottom 5% of normative references are presented.^28^

CCM, corneal confocal microscopy; IENFD, intraepidermal nerve fiber density; NBD, nerve branch density; NFD, nerve fiber density; NF, neurofibromatosis; NFL, nerve fiber length; NP_øTU_, neuropathic pain not caused by tumor; P_TU_, neuropathic pain caused by tumor; P_øNP_, pain other than neuropathic; øP, no pain; SWN-NF2, NF2-related schwannomatosis; SWN-NOS, schwannomatosis—not otherwise specified.

**Figure 5.**
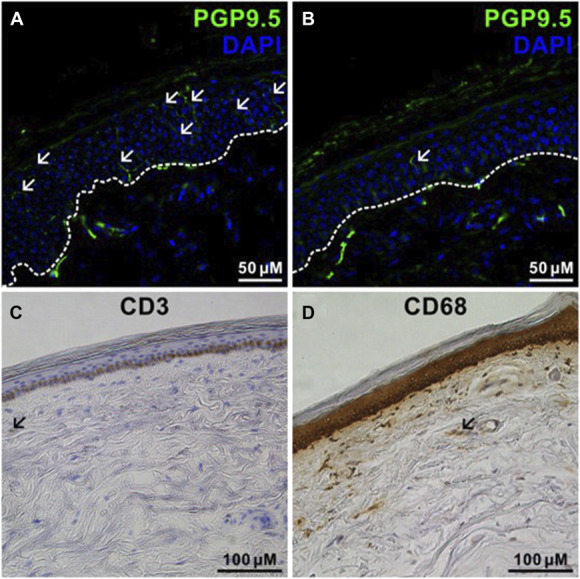
Skin innervation and inflammatory cell population. (A) + (B) Immunofluorescence staining with antibodies against PGP 9.5 and DAPI for determination of IENFD. (A) Normal IENFD; (B) reduced IENFD; (C) + (D) DAB staining for CD3 and CD68 for determination of intraepidermal T cells and macrophages. DAPI, 4,6-diamidin-2-phenylindol; DAB, 3,3-diaminobenzidin; IENFD, intraepidermal nerve fiber density; PGP, protein-gene product 9.5.

Skin-punch biopsy was performed from a distal and proximal site in 50 of 51 (98%) patients and only distally in 2 patients. There was a reduction in distal IENFD in 26 of 50 patients (52%) with NF: 15 of 32 (47%) with NF1, 7 of 11 (67%) with SWN-NF2, and 4 of 7 (57%) with SWN-NOS. Proximal IENFD was reduced in 11 of 48 patients (23%) with NF: 6 of 31 (19%) with NF1, 4 of 11 with SWN-NF2 (36%), and 1 of 6 (17%) with SWN-NOS (Table 3). Intraepidermal nerve fiber density was lower in patients suffering from neuropathic pain without tumor association (Fig. 5).

**Figure 6.**
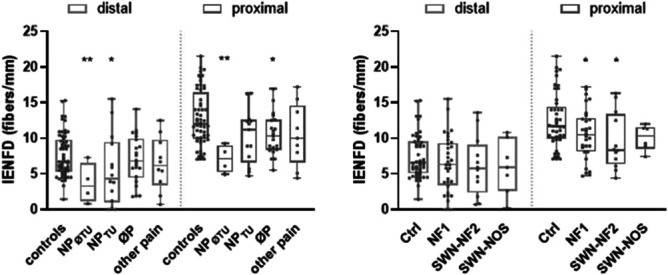
Quantitative analysis of distal and proximal IENFD. IENFD of the distal and proximal leg stratified for (A) pain type and (B) NF type. Reduced IENFD in patients suffering from neuropathic pain with no tumor association. IENFD, intraepidermal nerve fiber density; NF, neurofibromatosis; NP, neuropathic pain; NP_øTU_, neuropathic pain not caused by tumor; NP_TU_, neuropathic pain caused by tumor; P, pain; P_øNP_, pain other than neuropathic; øP, no pain; SWN-NOS, schwannomatosis—not otherwise specified, NF2-related schwannomatosis (SWN-NF2).

There was a generalized decrease of skin innervation in 9 of 50 (18%) patients with NF. Histological assessment did not reveal cellular infiltration of dermal T cells and macrophages (Fig. 6).

### 3.4. Alterations in nerve structure and function in neurofibromatosis patients

Paresis, tactile sensory loss, and/or diminished reflexes were present in 18 of 51 (35%) patients with NF. Nerve conduction studies were performed in 41 of 51 (80%) patients and 8 of 41 (20%) showed axonal injury, whilst 4 of 41 (10%) patients had mixed demyelination and axonal injury.

High-resolution ultrasonography was performed in 33 of 51 (65%) patients. CSA, as measured by nerve ultrasound, reflects the overall nerve structure, including the connective tissue and smaller nerve fibers, in addition to large fibers. We identified focal CSA enlargement in 10 of 33 (30%) patients and generalized caliber thickening in 9 of 33 (27%) patients (Table 5). The observed abnormalities did not follow a pattern typical of polyneuropathy, and there was no correlation between structural morphological abnormalities in sonography and findings in electrophysiology.

**Table 5:** Sonography data of the median nerve.

	Total	NF/SWN type		Pain subgroup	
Sonography performed	33/51 (65%)	NF1 SWN-NF2 SWN-NOS	24/33 (73%) 5/11 (45%) 4/7 (57%)	NP_øTU_ NP_TU_ P_øNP_ øP	4/4 (100%) 7/14 (50%) 6/10 (60%) 16/23 (70%)
Focal enlargement	10/33 (30%)	NF1 SWN-NF2 SWN-NOS	7/24 (29%) 3/5 (60%) 0/4	NP_øTU_ NP_TU_ P_øNP_ øP	1/4 (25%) 3/7 (43%) 2/6 (33%) 4/16 (25%)
Generalized enlargement		NF1 SWN-NF2 SWN-NOS	7/24 (29%) 2/5 (40%) 0/4	NP_øTU_ NP_TU_ P_øNP_ øP	0/4 2/7 (29%) 2/6 (33%) 5/16 (31%)

NF, neurofibromatosis; NP_øTU_, neuropathic pain not caused by tumor; P_TU_, neuropathic pain caused by tumor; P_øNP_, pain other than neuropathic; øP, no pain; SWN, schwannomatosis; SWN-NF2, NF2-related schwannomatosis; SWN-NOS, schwannomatosis—not otherwise specified.

### 3.5. Elevated perception thresholds and skin denervation in patients with neuropathic pain

We investigated whether patients reporting neuropathic pain showed evidence of SFI and whether the presence of SFI influenced the presence or intensity of neuropathic pain. The diagnosis of SFI was based on a combination of individual pain history, neurological examination, QST data, and skin-punch biopsy. To fulfill the diagnostic criteria for SFI,^7^ at least 2 pathological findings were required. Pathological findings included QST abnormalities (such as altered mechanical pain threshold, cold detection threshold, or thermal sensory limen), as well as a reduced IENFD in the skin biopsy. Tables 6 and 7 provide an overview of the patients classified as SFI.

**Table 6:** Patients with NF or SWN with small fiber impairment.

Patient ID	Age (yrs)	Sex	NF/SWN type	Pain location	QST	IENFD distal/proximal (fibers/mm)
NF01	55	M	SWN-NF2	Whole body	Elevated CDT	0.8/6.1
NF24	57	M	1	Neck, abdomen	Normal	1.2/5.3
NF28	42	M	1	Shoulders, neck, legs	Elevated CDT	2.3/4.9
NF41	44	F	1	Right arm, legs, back	Elevated CDT, WDT, TSL	3.8/16.2
NF43	60	F	1	Back, legs	Normal	4.
NF48	43	M	SWN-NOS	Right leg	Normal	2.6/12
NF50	46	F	SWN-NOS	Right leg	Elevated CDT, WDT, TSL	0.2/7.4

CDT, cold detection threshold; F, female; IENFD, intraepidermal nerve fiber density; M, male; NF, neurofibromatosis; QST, quantitative sensory testing; SWN, schwannomatosis; SWN-NF2, NF2-related schwannomatosis; SWN-NOS, schwannomatosis—not otherwise specified; WDT, warm detection threshold.

**Table 7:** CCM data in patients with small fiber impairment.

Patient ID	Age (yrs)	Sex	NF/SWN type	NFD (fibers/mm)	NBD (fibers/mm)	NFL (mm/mm)
NF01	55	M	SWN-NF2	9.4	11.5	4.9
NF24	57	M	1	14.6	26.1	9.2
NF28	42	M	1	27.1	73.96	17.7
NF41	44	F	1	33.3	77.1	18.6
NF43	60	F	1	15.6	36.5	13.1
NF48	43	M	SWN-NOS	26.1	35.4	13.7
NF50	46	F	SWN-NOS	42.7	76	21.9

CCM, corneal confocal microscopy; F, female; IENFD, intraepidermal nerve fiber density; M, male; NBD, nerve branch density; NFD, nerve fiber density; NFL, nerve fiber length; NF, neurofibromatosis; SFP, small fiber pathology; SWN, schwannomatosis; SWN-NF2, NF2-related schwannomatosis; SWN-NOS, schwannomatosis—not otherwise specified.

Among the patients with neuropathic symptoms, 12 of 18 (67%) exhibited sensory deficits, with hypesthesia observed in 10 patients and tingling paresthesias in 2 patients. Only 1 of 18 (6%) patients experienced motor deficits, which were mild paresis of the lower extremities. None of the 18 patients suffered from acral, burning pain, typical for SFN. In 14 of 18 (78%) patients, the pain was attributable to a nerve tumor (NP_TU_).

Individual QST profiles of patients with neuropathic pain in comparison with published normative values^16^ revealed elevated thermal perception thresholds in 5 of 18 (28%) patients, indicating small nerve fiber dysfunction.

In skin-punch biopsies, 12 of 18 (67%) patients showed skin denervation with the following patterns: 12 of 18 (67%) distal reduction, 6 of 18 (33%) proximal reduction, and 6 of 18 (33%) proximal and distal reduction of IENFD (Table 4).

Corneal confocal microscopy showed a reduction in NFL in 3 of 18 (17%) patients, and NFD and NBD were additionally pathological in 1 of 18 (6%) and NBD in 2 of 18 (11%) patients (Table 3).^29^ Hence, 7 of 18 (39%) patients with neuropathic pain also fulfilled the current diagnostic criteria of SFI.^5^ The distribution among the patient groups was 4 NF1, 1 SWN-NF2, and 2 SWN-NOS.

## 4. Discussion

In this single-center, tertiary care setting, we have established the frequency and characteristics of pain in patients with NF, with a particular focus on neuropathic pain, both related and unrelated to tumors. Pain is a common and significant symptom in patients with NF,^34^ impacting their health-related quality of life.^34^ In our cohort, 55% of patients reported pain, with 35% experiencing neuropathic pain, of which 14 of 18 (78%) cases were tumor-related, while 4 of 18 (22%) were unrelated to tumor involvement. Furthermore, 7 of 18 (39%) patients with neuropathic pain showed signs of small fiber impairment. However, it is important to note that none of these patients reported the acral, burning pain typical of SFN. Instead, our findings suggest that while SFI was observed in a subset of patients, the clinical presentation did not fully align with the typical SFN phenotype. These results should be interpreted with caution, given the limitations of a single-center study.

Neuropathic pain is often described as the hallmark symptom in SWN-NOS^8^; however, we also observed neuropathic pain in patients with NF1 and SWN-NF2. Pain was associated with a compressing nerve tumor in 14 of 18 (78%) of our patients. However, 4 of 18 (22%) patients suffered from neuropathic pain without an underlying morphological basis in imaging studies. These findings are summarized in Table 1, highlighting the distinction between tumor-related and non-tumor-related neuropathic pain. We show that pain may be associated with SFI, which may be attributed to neuroinflammation and altered secretion of factors from diseased Schwann cells that alter nociception.^19,21^ This may explain why only a subset of patients with tumors develop neuropathic pain. Indeed, we found evidence of SFI in one-third of patients with NPTU. Notably, this dysfunction was observed beyond the territory of the tumor, as demonstrated by abnormal findings in CCM and skin-punch biopsy, suggesting a generalized small fiber involvement. While endoneurial tumorous microlesions may contribute to neuropathic pain in tumor-related cases,^10^ the presence of SFI in patients with NPTU indicates that small fiber dysfunction can occur independently of tumor-related nerve lesions. This highlights the complexity of small fiber involvement in a disease primarily characterized by focal nerve pathology.

In patients with SWN-NF2, microlesions within peripheral nerves have been described; whereas, in NF1, these alterations are frequently categorized as plexiform tumors.^30,33^ Notably, abnormalities in median nerve ultrasonography were observed in all patients with SWN-NF2 examined and two-thirds of those with NF1. However, there was no association between pain and focal or general nerve enlargement, and there was no functional impairment in the median nerve innervation area in our cohort.

Interestingly, QST in the feet identified only mild alterations in thermal detection thresholds, which contrasts with the more pronounced distal abnormalities in patients with diabetic polyneuropathy.^27^ Thus, in NF, SFI may exhibit a more scattered and non-length-dependent distribution, with minimal abnormality in QST profiles at the distal area on the dorsal foot. Furthermore, we also show differences in relation to reduced corneal innervation in patients with NF with a reduction in 19% of those with NF1, 55% of those with SWN-NF2, and none of the patients with SWN-NOS. A study of 51 patients with NF1 showed that whilst only 8% had abnormal nerve conduction, thermal thresholds were abnormal in 13%, IENFD was reduced in 22%, and corneal NFL was pathologically reduced in 52%.^2^ Another recent study has also shown that patients with NF1 have decreased corneal sensitivity and increased corneal nerve branching but with no changes in tear levels of nerve growth factor or brain-derived neurotrophic factor levels.^17^

Small nerve fiber dysfunction in patients with reduced IENFD was reported as a distinct characteristic of SWN and was found in approximately half of our cohort with SWN-NF2.^9,10^ A reduction in IENFD was most common in SWN-NF2 in our cohort and occurred in approximately half of the patients with NF1 and SWN-NOS. It is noteworthy that the reduction in IENFD was independent of age or tumor localization. Furthermore, the reduction of IENFD, particularly in patients with neuropathic pain without a tumor, suggests that SFI, rather than tumor compression may contribute to pain.

We demonstrate abnormalities in morphology and electrophysiology of the large fibers in one-third of patients with NF. It is worth noting that despite the presence of large tumors or nerves completely infiltrated by tumors, functional impairment was rare, especially in patients with NF1. However, the small sample size and the considerable data variability limit our potential for generalizable conclusions and subgroup analysis, especially as some severely impaired patients were excluded due to a lack of physical fitness. We present these findings recognizing that further validation in larger, multicenter cohorts is needed to confirm and expand upon these observations.

Our study provides important insights into the prevalence of pain among the different NF subgroups and its impact on patients' health-related quality of life. It also demonstrates that neuropathic pain was primarily associated with the presence of tumors, but also occurred in connection with SFI.

## Disclosures

The authors have no conflict of interest to declare.
